# NONINVASIVE MANAGEMENT OF INTRACTABLE HICCUPS

**DOI:** 10.51894/001c.123073

**Published:** 2024-08-30

**Authors:** Derek Bowman

**Affiliations:** 1 College of Osteopathic Medicine Michigan State University

## 72

### INTRODUCTION

Singultus, commonly known as hiccups, can be broken down into several categories: acute (less than 48 hours), persistent (over 2 days), and intractable (over 1 month). While acute singultus typically resolves idiopathically, persistent and intractable singultus pose significant challenges in terms of diagnosis and management. Current understanding suggests singultus arises from irritations along a reflex arc involving the vagus and phrenic nerves, though the exact mechanism remains elusive. Moreover, various etiologies, including organic, psychogenic, and idiopathic factors, contribute to intractable singultus, complicating diagnosis and treatment. Intractable singultus, in particular, can lead to discomfort, emotional disturbance, and potential complications such as dehydration and metabolic derangements.

Treatment strategies for singultus encompass pharmacologic and non-pharmacologic approaches; however, the efficacy of pharmacotherapy varies, and medications may entail significant side effects. Non-pharmacologic interventions, including phrenic nerve ablation and microvascular decompression, remain understudied and are typically considered after exhausting non-invasive options.

### CASE DESCRIPTION

One emerging avenue for singultus management is osteopathic manipulative treatment (OMT), which aims to alleviate somatic dysfunction and improve nervous system function. While literature on OMT in singultus treatment is limited, with only four published case reports, it highly suggests its potential benefits. The most recent case in the literature highlights beneficial intractable singultus management with OMT in a 23-year-old woman. Building upon that research, our case series highlights the use of OMT in two more female patients in their twenties with intractable singultus, where a structured treatment protocol was followed and outcomes were monitored seven days pre and post-treatment. The treatment consisted of myofascial release, muscle energy technique, and counterstrain to areas with tissue texture abnormalities in an attempt to reduce intractable singultus.

### DISCUSSION/CONCLUSIONS

In our study, OMT demonstrated promising results, with patients experiencing a reduction in hiccup frequency following treatment, echoing the results of the previous case study in the literature. This underscores the potential of OMT as a strong non-pharmacologic option for singultus management, especially in cases where conventional therapies have been ineffective or poorly tolerated. Further research is warranted to elucidate the mechanisms underlying OMT’s efficacy and establish its role in the broader spectrum of intractable singultus management.

